# Ischemic Gastropathy Treated with Celiac Artery Revascularization

**DOI:** 10.7759/cureus.5949

**Published:** 2019-10-21

**Authors:** Jeff Anucha, Jonathan Pinto, Joan Culpepper-Morgan, Alvaro Genao, Neil Resnick

**Affiliations:** 1 Internal Medicine, Columbia University School of Physicians and Surgeons at Harlem Hospital Center, New York, USA; 2 Internal Medicine: Gastroenterology, Columbia University School of Physicians and Surgeons at Harlem Hospital Center, New York, USA; 3 Radiology, Columbia University School of Physicians and Surgeons at Harlem Hospital Center, New York, USA

**Keywords:** stomach, ischemia, celiac artery stenosis, upper gi bleed, ir-guided stenting

## Abstract

Ischemic gastropathy is an uncommon diagnosis due to extensive arterial collaterals that supply the stomach. The mucosal integrity of the stomach is dependent upon this redundant circulation. Hence, its diagnosis is infrequently entertained in patients presenting with an upper gastrointestinal (GI) bleed. Herein, we report a case of a 76-year-old woman with hypertension, hyperlipidemia, and chronic kidney disease on dialysis who developed an upper GI bleed after becoming septic and hypotensive.

## Introduction

The stomach has a rich and redundant blood supply and is the only organ to receive arterial supply from all three branches of the celiac trunk. This is achieved through five major direct arterial sources, which are the right and left gastric, the right and left gastroepiploic, and the short gastric arteries. These form a complex and extensive plexus of collaterals that makes the stomach less prone to ischemic injury [1-2]. The mucosal integrity of the stomach is dependent upon this redundant circulation as well. Ischemic gastropathy as a direct result of mesenteric ischemia has been reported but is an uncommon diagnosis; hence, it is rarely entertained in patients presenting with an upper gastrointestinal (GI) bleed. We report a case of a 76-year-old woman with hypertension, hyperlipidemia, and chronic kidney disease on dialysis who developed an upper gastrointestinal bleed after she became septic and hypotensive.

Part of this article has been presented earlier (Pinto J, Culpepper-Morgan J, Anucha JC, Genao A, Resnick N. P2602 - A rare case of severe ischemic gastropathy causing upper GI bleed. ACG 2018 Annual Scientific Meeting Abstracts. American College of Gastroenterology; https://eventscribe.com/2018/ACG/ajaxcalls/PosterInfo.asp?PosterID=161883&efp=RFNSWFFHSFY2NDI0&rnd=0.604323).

## Case presentation

A 76-year-old woman with hypertension, hyperlipidemia, and chronic kidney disease presented with acute shortness of breath. She was tachycardic, tachypneic, and hypotensive on exam, with expiratory wheezes and abdominal distention. Laboratory findings included elevated lactate, metabolic acidosis, leukocytosis of 22 K/uL, and hemoglobin of 10.7 g/dl. She was managed for acute respiratory failure and septic shock. Despite the treatment of her sepsis, lactic acidosis persisted.

**Figure 1:**
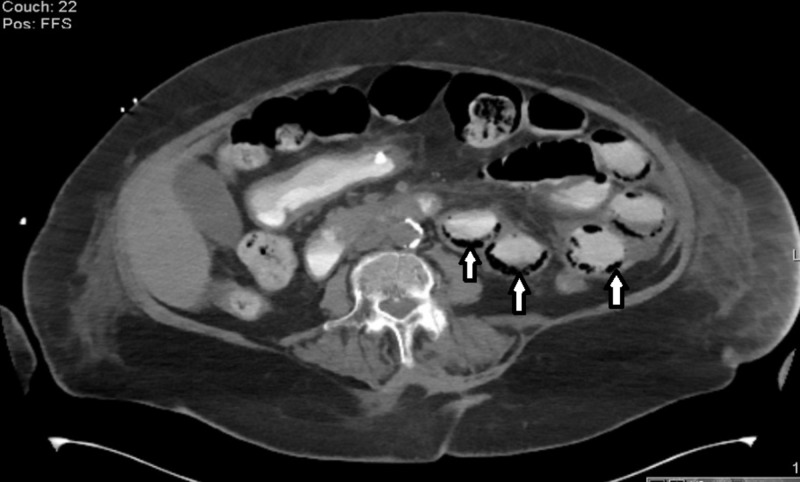
Pneumatosis intestinalis; air within the bowel wall (arrows)

An abdominal computerized tomography (CT), which was obtained to evaluate for ongoing abdominal distention, revealed pneumatosis intestinalis (Figure 1).

**Figure 2:**
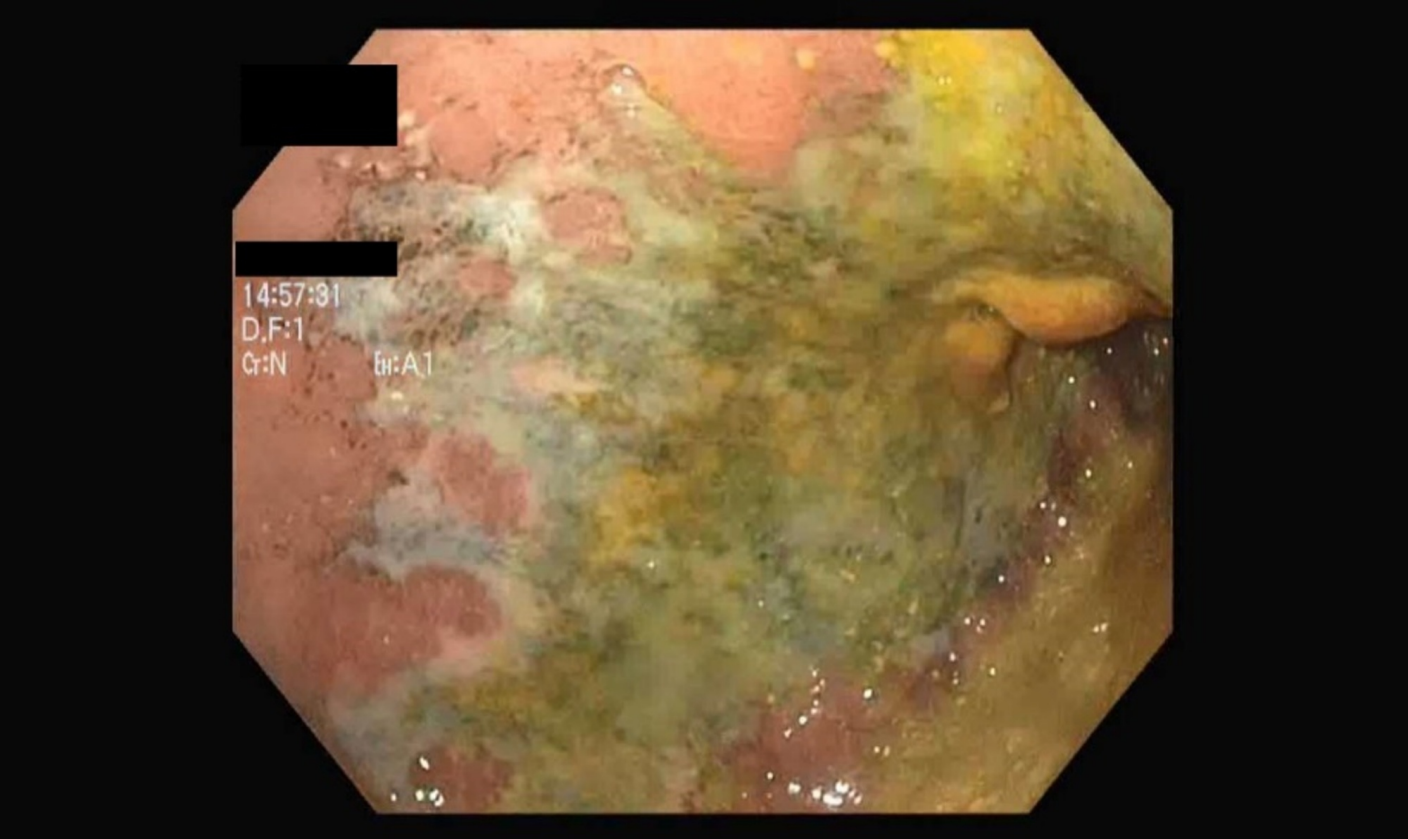
Shallow ischemic anterior gastric ulcer

Metronidazole intravenous (IV) was then started for the management of gas-forming organisms, which led to an immediate improvement in leukocytes from 24 K/uL to 8.6 K/uL. The patient developed melena and anemia (Hgb 6.4g/dl) afterwards. Esophagogastroduodenoscopy (EGD) revealed extensive shallow ischemic ulcers involving most of the anterior gastric wall and denuded duodenal mucosa (Figure 2).

**Figure 3:**
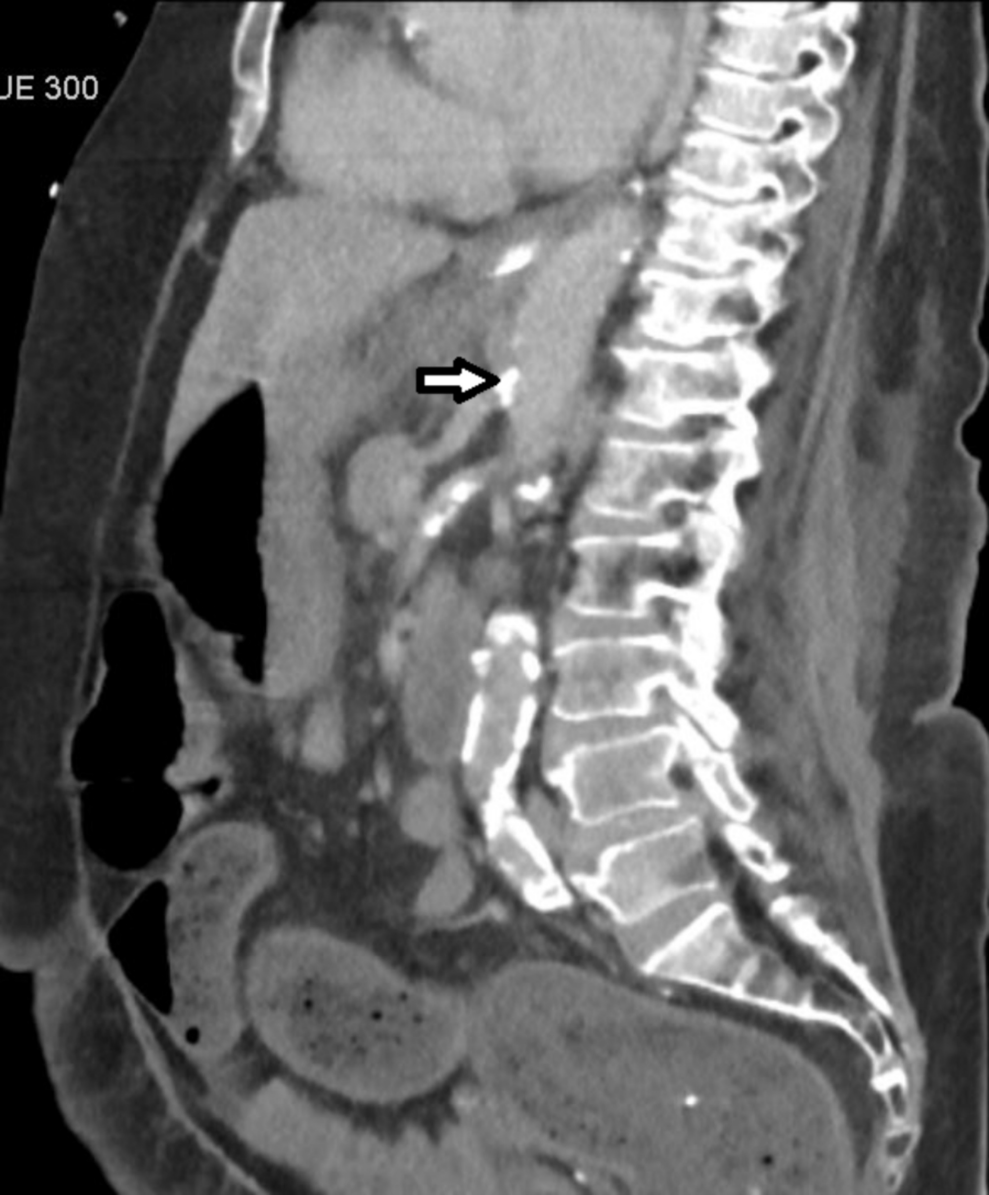
Atherosclerotic plaque within the celiac artery (arrow) and SMA causing high-grade stenosis SMA: superior mesenteric artery

Subsequent CT angiography of the abdomen showed marked atherosclerotic plaques causing high-grade stenosis of the celiac axis, lesser stenosis of the superior mesenteric artery, and complete resolution of pneumatosis intestinalis (Figure 3).

**Figure 4:**
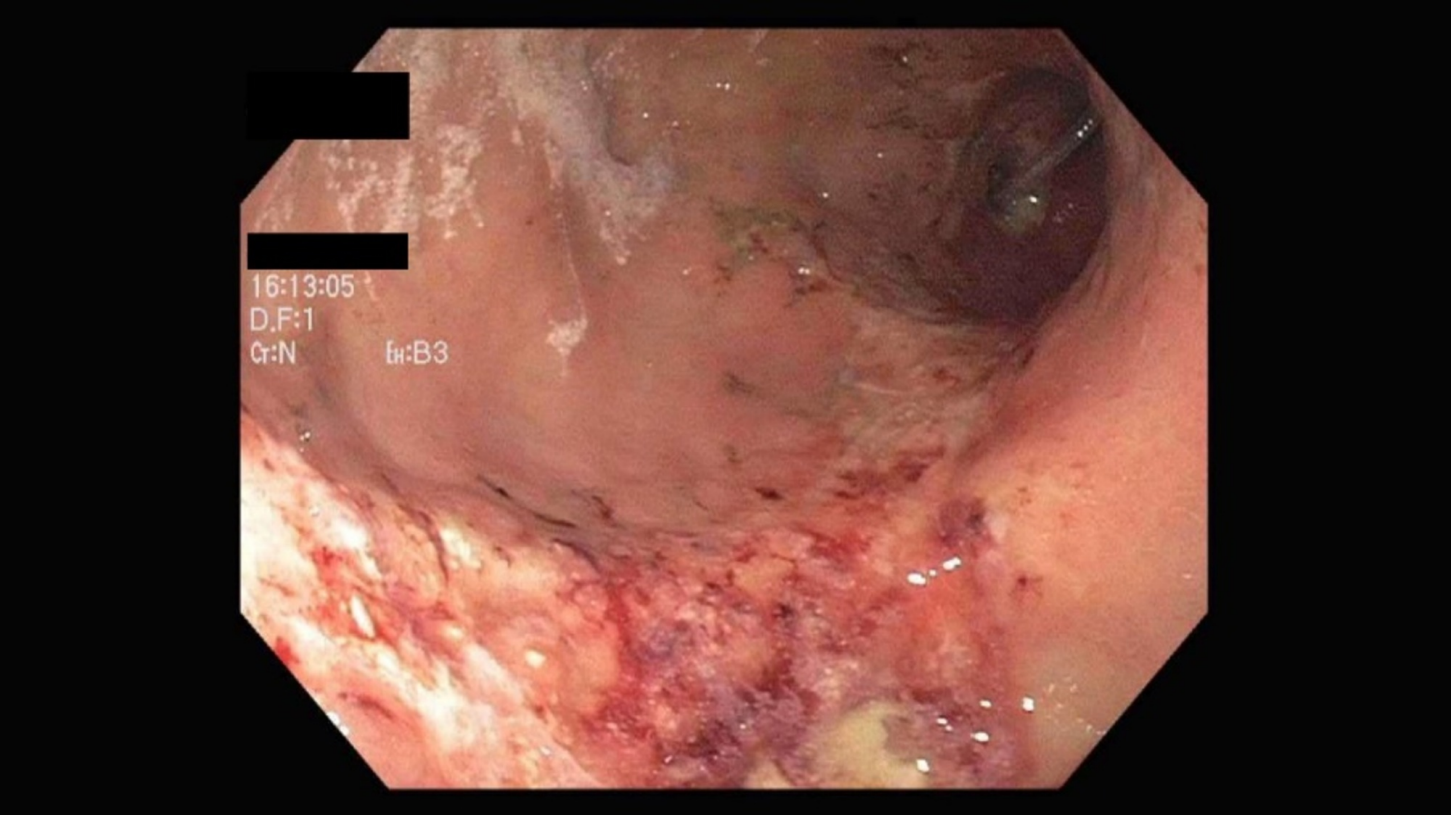
Resolution of the ischemic gastric ulcer after celiac artery stenting

An interventional radiology (IR)-guided stent was placed across the celiac artery with successful reperfusion confirmed by fluoroscopy. Serum lactate levels rapidly normalized and a repeat EGD three weeks later showed near-complete resolution of gastric ulcers (Figure 4).

## Discussion

Ischemic gastropathy is a rare cause of upper GI bleeding because of an extensive network of vascular collaterals that supplies the stomach wall [1-2]. Although stenosis of the celiac trunk alone may be asymptomatic, concomitant superior mesenteric artery (SMA) stenosis may result in ischemic lesions in the stomach wall [3-5]. Clinical manifestations can include pain, nausea, vomiting, and bleeding. Gastric ischemia may be secondary to systemic hypoperfusion in the setting of shock, local splanchnic vessel stenosis, or thrombosis. Subsequent ischemia leads to gastric mucosal barrier dysfunction, accumulation of gastric acid, and eventual ulceration.

There were several predisposing risk factors to gastric ischemia in our patient, including diabetes, hypertension, and hypercholesterolemia [6]. Episodes of global hypoperfusion from sepsis may have contributed to a low flow state as well. She was on diuretics, which has been reported as a possible contributing factor, as well as intermittent hemodialysis, which has been shown to cause accelerated rates of atherosclerotic plaques [7-8]. In previous case reports reviewed, the diagnosis of gastric ischemia was often delayed because of the time spent on eliminating more common conditions, whilst a lack of response to maximal antacid therapy was observed [9-11]. For the gastrointestinal endoscopist, there should be a high index of suspicion when there are gastric ulcers in patients at risk for vascular disease [12].

The presence of SMA stenosis likely also contributed to small bowel ischemia, resulting in pneumatosis intestinalis. Correction of her underlying sepsis led to an improvement of bowel ischemia findings on follow-up CT of the abdomen.

While ischemia secondary to transient low flow states may be managed conservatively with bowel rest and broad-spectrum antibiotics, definitive recanalization of the celiac trunk may be required when high-grade stenosis is present. This can be achieved either surgically or via percutaneous transluminal intervention, with the latter preferred in high-risk patients as in our case.

## Conclusions

Although exceedingly rare, ischemic gastropathy can occur and is potentially treatable with carefully selected revascularization therapy in high-risk patients.
